# 4a-Hy­droxy-9-(2-meth­oxy­phen­yl)-4,4a,5,6,7,8,9,9a-octa­hydro-3*H*-xanthene-1,8(2*H*)-dione

**DOI:** 10.1107/S1600536810050191

**Published:** 2010-12-04

**Authors:** Wan-Sin Loh, Hoong-Kun Fun, B. Palakshi Reddy, V. Vijayakumar, S. Sarveswari

**Affiliations:** a X-ray Crystallography Unit, School of Physics, Universiti Sains Malaysia, 11800 USM, Penang, Malaysia; b Organic Chemistry Division, School of Advanced Sciences, VIT University, Vellore 632 014, India

## Abstract

In the title compound, C_20_H_22_O_5_, an *S*(6) ring motif is formed by an intra­molecular C—H⋯O hydrogen bond, which contributes to the stabilization of the mol­ecule. In the xanthene system, the cyclo­hexane ring adopts a chair conformation, the cyclo­hexene ring adopts a half-boat conformation and the tetra­hydro­pyran ring adopts a half-chair conformation. The mean plane of the four essentially planar atoms of the tetra­hydro­pyran ring [r.m.s deviation = 0.092 (1) Å] forms a dihedral angle of 64.13 (6)° with the mean plane of the meth­oxy­phenyl group. In the crystal, inter­molecular O—H⋯O and weak C—H⋯O hydrogen bonds link mol­ecules into chains along the *a* axis, which are further stabilized by C—H⋯π inter­actions.

## Related literature

For background to and the biological activity of xanthenes and their derivatives, see: Menchen *et al.* (2003 ▶); Saint-Ruf *et al.* (1972 ▶); Ion *et al.* (1998 ▶); Knight & Stephens (1989 ▶); Jonathan *et al.* (1988 ▶). For ring conformations, see: Cremer & Pople (1975 ▶). For hydrogen-bond motifs, see: Bernstein *et al.* (1995 ▶). For standard bond-length data, see: Allen *et al.* (1987 ▶). For a related structure, see: Reddy *et al.* (2009 ▶). For the stability of the temperature controller used in the data collection, see: Cosier & Glazer (1986 ▶).
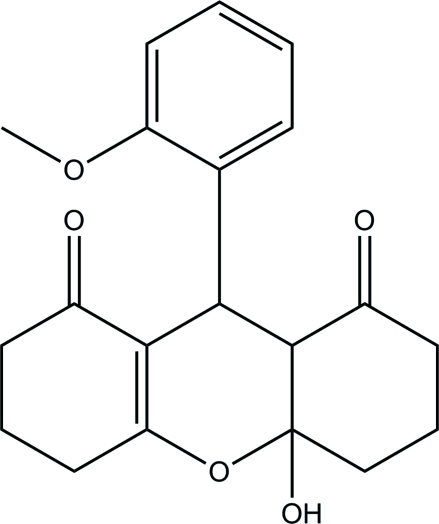


## Experimental

### 

#### Crystal data

C_20_H_22_O_5_
*M* *_r_* = 342.38
Triclinic, 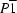

*a* = 7.1060 (1) Å
*b* = 7.8897 (1) Å
*c* = 15.1001 (2) Å
α = 91.285 (1)°
β = 101.251 (1)°
γ = 101.129 (1)°
*V* = 813.10 (2) Å^3^
*Z* = 2
Mo *K*α radiation
μ = 0.10 mm^−1^
*T* = 100 K
0.44 × 0.23 × 0.10 mm

#### Data collection

Bruker SMART APEXII CCD area-detector diffractometer
Absorption correction: multi-scan (*SADABS*; Bruker, 2009) ▶ *T*_min_ = 0.957, *T*_max_ = 0.990
21213 measured reflections
4715 independent reflections
4132 reflections with *I* > 2σ(*I*)
*R*_int_ = 0.025

#### Refinement

*R*[*F* ^2^ > 2σ(*F* ^2^)] = 0.038
*wR*(*F* ^2^) = 0.110
*S* = 1.05
4715 reflections
231 parameters
H atoms treated by a mixture of independent and constrained refinement
Δρ_max_ = 0.46 e Å^−3^
Δρ_min_ = −0.25 e Å^−3^

### 

Data collection: *APEX2* (Bruker, 2009 ▶); cell refinement: *SAINT* (Bruker, 2009 ▶); data reduction: *SAINT*; program(s) used to solve structure: *SHELXTL* (Sheldrick, 2008) ▶; program(s) used to refine structure: *SHELXTL*; molecular graphics: *SHELXTL*; software used to prepare material for publication: *SHELXTL* and *PLATON* (Spek, 2009 ▶).

## Supplementary Material

Crystal structure: contains datablocks global, I. DOI: 10.1107/S1600536810050191/lh5179sup1.cif

Structure factors: contains datablocks I. DOI: 10.1107/S1600536810050191/lh5179Isup2.hkl

Additional supplementary materials:  crystallographic information; 3D view; checkCIF report

## Figures and Tables

**Table 1:** Hydrogen-bond geometry (Å, °) *Cg*1 is the centroid of the C14–C19 ring.

*D*—H⋯*A*	*D*—H	H⋯*A*	*D*⋯*A*	*D*—H⋯*A*
O5—H1*O*5⋯O3^i^	0.87 (2)	1.93 (2)	2.7877 (11)	166.3 (18)
C6—H6*A*⋯O4	0.98	2.32	2.9266 (12)	120
C16—H16*A*⋯O5^ii^	0.93	2.53	3.4172 (13)	160
C20—H20*B*⋯*Cg*1^ii^	0.96	2.67	3.5206 (13)	147

Symmetry codes: (i) 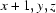; (ii) 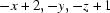.

**Fig. 1.**
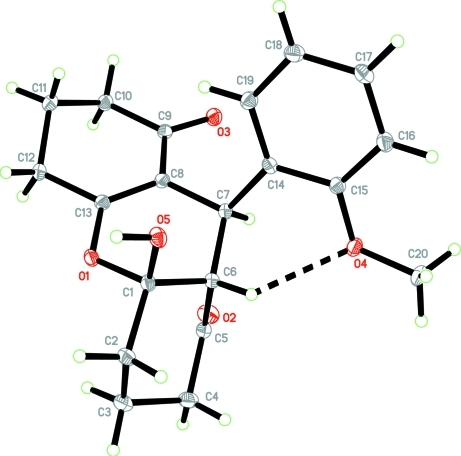
The molecular structure of the title compound, showing 50% probability displacement ellipsoids and the atom-numbering scheme. The dashed line indicates the intramolecular hydrogen bond.

**Fig. 2.**
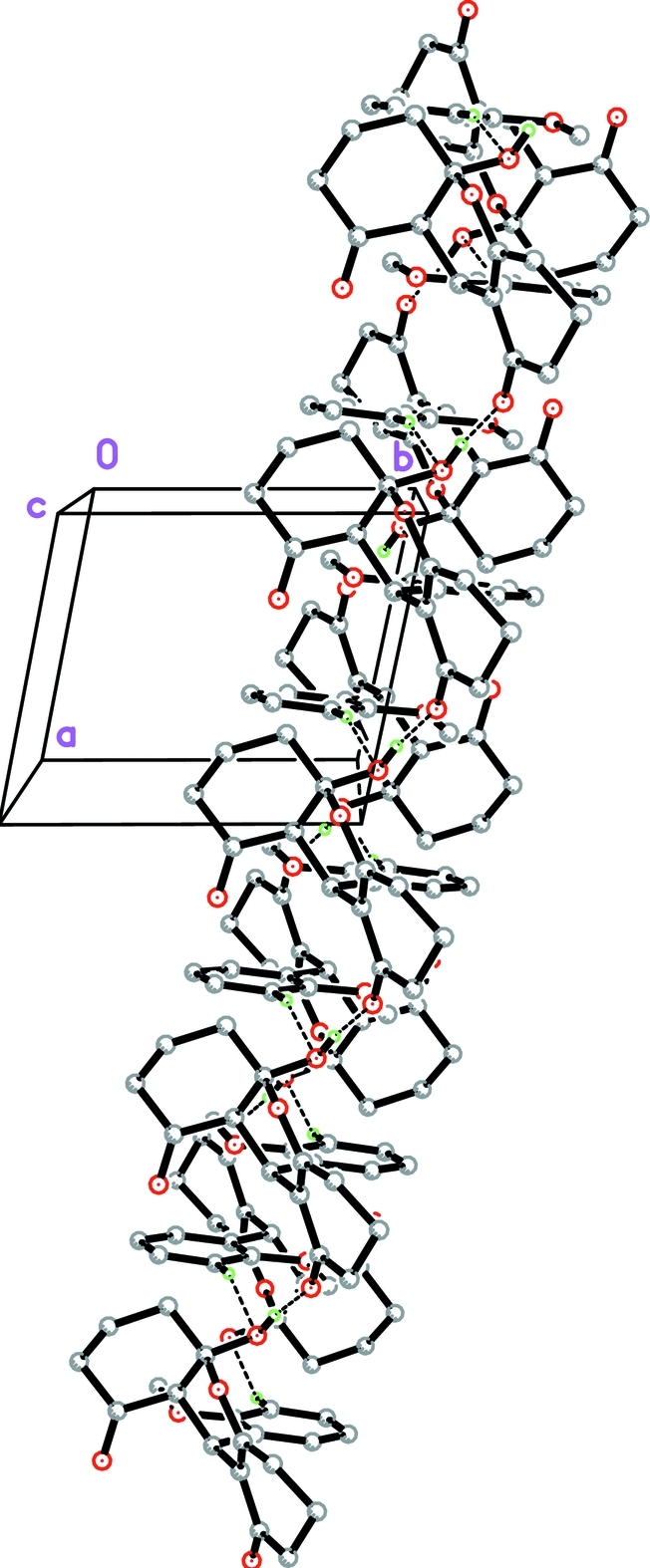
The crystal packing of the title compound, viewed along the c axis, showing a chain along the a axis. H atoms not involved in the intermolecular interactions (dashed lines) have been omitted for clarity.

## supplementary crystallographic information

### Comment

Xanthene derivatives are very important heterocyclic compounds and due to their useful spectroscopic properties, they have been widely used as dyes, fluorescent materials for visualization of bio-molecules and in laser technologies (Menchen *et al.*, 2003; Saint-Ruf *et al.*, 1972; Ion *et al.*, 1998). They have been reported for their agricultural bactericide activity, photodynamic therapy, antiflammatory effect and antiviral activity (Knight & Stephens, 1989; Jonathan *et al.*, 1988). Due to their wide range of applications, these compounds have received a great deal of attention in connection with their synthesis. In the synthesis of these compounds, intermediates play a key role, because these compounds can be easily converted into acridines and other biological active compounds.

In the title compound, an intramolecular C6—H6A···O4 hydrogen bond (Table 1) contributes to the stabilization of the molecule (Fig. 1), forming an *S*(6) ring motif (Bernstein *et al.*, 1995). The xanthene ring system consists of three rings which adopt different conformations. The cyclohexane ring (C1–C6) adopts a chair conformation with the puckering parameters Q = 0.5427 (11) Å, Θ = 4.67 (12)°, φ = 169.6 (15)° (Cremer & Pople, 1975). The cyclohexene ring (C8–C13) and the tetrahydropyran ring (O1/C1/C6/C7/C8/C13) adopt half-boat and half-chair conformations, with the puckering parameters, Q = 0.4831 (11) Å, Θ = 61.06 (13)°, φ = 176.13 (15)° and Q = 0.4497 (10) Å, Θ = 47.24 (13)°, φ = 87.44 (17)° (Cremer & Pople, 1975), respectively. The mean plane of the essentially planar atoms of the tetrahydropyran ring (C7/C8/C13/O1) [r.m.s deviation = 0.092 (1) Å] forms a dihedral angle of 64.13 (6)° with the methoxyphenyl group (C14–C20/O4). The bond lengths (Allen *et al.*, 1987) and angles are within the normal range and are comparable to the related structure (Reddy *et al.*, 2009).

In the crystal packing (Fig. 2), intermolecular O5—H1O5···O3^i^ and C16—H16A···O5^ii^ hydrogen bonds (see Table 1 for symmetry codes) link molecules into chains along the *a* axis which are further stabilized by C—H···*Cg*1^ii^ interactions (Table 1), involving C14–C19 ring.

### Experimental

A mixture of 2-methoxybenzaldehyde (0.365 ml, 0.0025 mol) and 1,3-cyclohexanedione (0.56 g, 0.005 mol) was refluxed in acetonitrile for 3 h. The progress of the reaction was monitored by TLC. After completion of the reaction, it was kept for 2 days for solid formation. The pure product was obtained by recrystallization of the crude product from ethanol. *M.p.*: 493–495 K, yield: 72%.

### Refinement

Atom H1O5 was located from the difference Fourier map and was refined freely [O–H = 0.874 (18) Å]. The remaining H atoms were positioned geometrically [C–H = 0.93 or 0.98 Å] and were refined using a riding model, with *U*_iso_(H) = 1.2 or 1.5 *U*_eq_(C). A rotating group model was applied to the methyl group.

### Crystal data

**Table:**

C_20_H_22_O_5_	*Z* = 2
*M**_r_* = 342.38	*F*(000) = 364
Triclinic, *P*1	*D*_x_ = 1.398 Mg m^−^^3^
Hall symbol: -P 1	Mo *K*α radiation, λ = 0.71073 Å
*a* = 7.1060 (1) Å	Cell parameters from 9956 reflections
*b* = 7.8897 (1) Å	θ = 2.6–37.2°
*c* = 15.1001 (2) Å	µ = 0.10 mm^−^^1^
α = 91.285 (1)°	*T* = 100 K
β = 101.251 (1)°	Block, colourless
γ = 101.129 (1)°	0.44 × 0.23 × 0.10 mm
*V* = 813.10 (2) Å^3^	

### Data collection

**Table:**

Bruker SMART APEXII CCD area-detector diffractometer	4715 independent reflections
Radiation source: fine-focus sealed tube	4132 reflections with *I* > 2σ(*I*)
graphite	*R*_int_ = 0.025
φ and ω scans	θ_max_ = 30.0°, θ_min_ = 2.6°
Absorption correction: multi-scan (*SADABS*; Bruker, 2009)	*h* = −9→9
*T*_min_ = 0.957, *T*_max_ = 0.990	*k* = −11→11
21213 measured reflections	*l* = −21→21

### Refinement

**Table:**

Refinement on *F*^2^	Primary atom site location: structure-invariant direct methods
Least-squares matrix: full	Secondary atom site location: difference Fourier map
*R*[*F*^2^ > 2σ(*F*^2^)] = 0.038	Hydrogen site location: inferred from neighbouring sites
*wR*(*F*^2^) = 0.110	H atoms treated by a mixture of independent and constrained refinement
*S* = 1.05	*w* = 1/[σ^2^(*F*_o_^2^) + (0.057*P*)^2^ + 0.3385*P*] where *P* = (*F*_o_^2^ + 2*F*_c_^2^)/3
4715 reflections	(Δ/σ)_max_ < 0.001
231 parameters	Δρ_max_ = 0.46 e Å^−^^3^
0 restraints	Δρ_min_ = −0.25 e Å^−^^3^

### Special details

**Table:**

Experimental. The crystal was placed in the cold stream of an Oxford Cryosystems Cobra open-flow nitrogen cryostat (Cosier & Glazer, 1986) operating at 100.0 (1) K.
Geometry. All e.s.d.'s (except the e.s.d. in the dihedral angle between two l.s. planes) are estimated using the full covariance matrix. The cell e.s.d.'s are taken into account individually in the estimation of e.s.d.'s in distances, angles and torsion angles; correlations between e.s.d.'s in cell parameters are only used when they are defined by crystal symmetry. An approximate (isotropic) treatment of cell e.s.d.'s is used for estimating e.s.d.'s involving l.s. planes.
Refinement. Refinement of *F*^2^ against ALL reflections. The weighted *R*-factor *wR* and goodness of fit *S* are based on *F*^2^, conventional *R*-factors *R* are based on *F*, with *F* set to zero for negative *F*^2^. The threshold expression of *F*^2^ > σ(*F*^2^) is used only for calculating *R*-factors(gt) *etc*. and is not relevant to the choice of reflections for refinement. *R*-factors based on *F*^2^ are statistically about twice as large as those based on *F*, and *R*- factors based on ALL data will be even larger.

### Fractional atomic coordinates and isotropic or equivalent isotropic displacement parameters (Å^2^)

**Table:**

	*x*	*y*	*z*	*U*_iso_*/*U*_eq_	
O1	0.99328 (11)	0.05712 (9)	0.14023 (5)	0.01184 (15)	
O2	0.70333 (12)	0.35590 (10)	0.19525 (6)	0.01849 (17)	
O3	0.34225 (11)	−0.15228 (10)	0.17249 (5)	0.01475 (16)	
O4	0.74431 (12)	0.15152 (9)	0.43898 (5)	0.01407 (16)	
O5	1.11520 (11)	−0.03057 (9)	0.28104 (5)	0.01311 (15)	
C1	1.06767 (15)	0.11229 (12)	0.23546 (6)	0.01072 (18)	
C2	1.24660 (15)	0.25412 (13)	0.23649 (7)	0.01350 (19)	
H2A	1.3340	0.2104	0.2039	0.016*	
H2B	1.3158	0.2835	0.2986	0.016*	
C3	1.19583 (16)	0.41749 (13)	0.19413 (7)	0.0163 (2)	
H3A	1.1438	0.3927	0.1298	0.020*	
H3B	1.3137	0.5066	0.2015	0.020*	
C4	1.04414 (17)	0.48393 (13)	0.23831 (8)	0.0172 (2)	
H4A	1.1005	0.5208	0.3012	0.021*	
H4B	1.0064	0.5825	0.2075	0.021*	
C5	0.86650 (16)	0.34065 (13)	0.23232 (7)	0.01310 (19)	
C6	0.90683 (15)	0.17311 (12)	0.27485 (6)	0.01074 (18)	
H6A	0.9570	0.2001	0.3398	0.013*	
C7	0.71877 (15)	0.03250 (12)	0.26338 (6)	0.01037 (18)	
H7A	0.6112	0.0933	0.2644	0.012*	
C8	0.67731 (15)	−0.05984 (12)	0.17062 (6)	0.01066 (18)	
C9	0.47820 (15)	−0.15295 (12)	0.13264 (6)	0.01103 (18)	
C10	0.43704 (15)	−0.24679 (13)	0.04013 (7)	0.01356 (19)	
H10A	0.3871	−0.1725	−0.0052	0.016*	
H10B	0.3363	−0.3499	0.0383	0.016*	
C11	0.61838 (16)	−0.29864 (13)	0.01678 (7)	0.01432 (19)	
H11A	0.6579	−0.3863	0.0563	0.017*	
H11B	0.5882	−0.3473	−0.0451	0.017*	
C12	0.78507 (16)	−0.14125 (13)	0.02780 (7)	0.01329 (19)	
H12A	0.9051	−0.1781	0.0224	0.016*	
H12B	0.7561	−0.0643	−0.0200	0.016*	
C13	0.81329 (15)	−0.04614 (12)	0.11791 (6)	0.01064 (18)	
C14	0.71718 (14)	−0.09100 (12)	0.33983 (6)	0.01062 (18)	
C15	0.72872 (15)	−0.02412 (12)	0.42848 (7)	0.01140 (18)	
C16	0.72200 (16)	−0.13289 (13)	0.49996 (7)	0.01399 (19)	
H16A	0.7337	−0.0869	0.5585	0.017*	
C17	0.69757 (16)	−0.31127 (13)	0.48290 (7)	0.0150 (2)	
H17A	0.6903	−0.3844	0.5301	0.018*	
C18	0.68398 (16)	−0.38035 (13)	0.39597 (7)	0.0146 (2)	
H18A	0.6674	−0.4992	0.3847	0.018*	
C19	0.69553 (15)	−0.26938 (13)	0.32551 (7)	0.01285 (19)	
H19A	0.6886	−0.3158	0.2675	0.015*	
C20	0.79471 (17)	0.22709 (13)	0.52994 (7)	0.0154 (2)	
H20A	0.8251	0.3509	0.5289	0.023*	
H20B	0.9066	0.1875	0.5625	0.023*	
H20C	0.6861	0.1934	0.5593	0.023*	
H1O5	1.201 (3)	−0.067 (2)	0.2552 (12)	0.027 (4)*	

### Atomic displacement parameters (Å^2^)

**Table:**

	*U*^11^	*U*^22^	*U*^33^	*U*^12^	*U*^13^	*U*^23^
O1	0.0112 (3)	0.0143 (3)	0.0096 (3)	−0.0001 (3)	0.0038 (3)	−0.0002 (2)
O2	0.0168 (4)	0.0169 (4)	0.0226 (4)	0.0053 (3)	0.0039 (3)	0.0048 (3)
O3	0.0130 (4)	0.0158 (3)	0.0160 (3)	0.0021 (3)	0.0054 (3)	−0.0011 (3)
O4	0.0207 (4)	0.0119 (3)	0.0100 (3)	0.0039 (3)	0.0036 (3)	−0.0005 (2)
O5	0.0144 (4)	0.0141 (3)	0.0132 (3)	0.0058 (3)	0.0053 (3)	0.0034 (3)
C1	0.0112 (4)	0.0117 (4)	0.0091 (4)	0.0014 (3)	0.0027 (3)	0.0007 (3)
C2	0.0111 (4)	0.0148 (4)	0.0141 (4)	−0.0001 (4)	0.0041 (3)	0.0004 (3)
C3	0.0160 (5)	0.0134 (4)	0.0194 (5)	−0.0004 (4)	0.0065 (4)	0.0020 (3)
C4	0.0175 (5)	0.0116 (4)	0.0229 (5)	0.0006 (4)	0.0076 (4)	0.0002 (4)
C5	0.0158 (5)	0.0115 (4)	0.0129 (4)	0.0022 (4)	0.0058 (4)	0.0000 (3)
C6	0.0114 (4)	0.0110 (4)	0.0098 (4)	0.0015 (3)	0.0030 (3)	0.0000 (3)
C7	0.0110 (4)	0.0109 (4)	0.0093 (4)	0.0018 (3)	0.0026 (3)	0.0003 (3)
C8	0.0119 (4)	0.0099 (4)	0.0099 (4)	0.0019 (3)	0.0021 (3)	0.0003 (3)
C9	0.0125 (4)	0.0097 (4)	0.0112 (4)	0.0026 (3)	0.0029 (3)	0.0014 (3)
C10	0.0132 (5)	0.0143 (4)	0.0122 (4)	0.0009 (4)	0.0025 (4)	−0.0024 (3)
C11	0.0161 (5)	0.0126 (4)	0.0140 (4)	0.0012 (4)	0.0044 (4)	−0.0025 (3)
C12	0.0156 (5)	0.0137 (4)	0.0108 (4)	0.0014 (4)	0.0052 (4)	−0.0016 (3)
C13	0.0119 (4)	0.0098 (4)	0.0099 (4)	0.0016 (3)	0.0021 (3)	0.0010 (3)
C14	0.0098 (4)	0.0121 (4)	0.0100 (4)	0.0017 (3)	0.0027 (3)	0.0014 (3)
C15	0.0106 (4)	0.0121 (4)	0.0118 (4)	0.0021 (3)	0.0032 (3)	0.0004 (3)
C16	0.0155 (5)	0.0157 (4)	0.0109 (4)	0.0028 (4)	0.0035 (4)	0.0014 (3)
C17	0.0162 (5)	0.0153 (4)	0.0139 (4)	0.0025 (4)	0.0039 (4)	0.0046 (3)
C18	0.0159 (5)	0.0119 (4)	0.0155 (4)	0.0020 (4)	0.0026 (4)	0.0017 (3)
C19	0.0130 (5)	0.0130 (4)	0.0122 (4)	0.0022 (4)	0.0022 (3)	−0.0003 (3)
C20	0.0189 (5)	0.0158 (4)	0.0112 (4)	0.0043 (4)	0.0020 (4)	−0.0025 (3)

### Geometric parameters (Å, °)

**Table:**

O1—C13	1.3529 (12)	C8—C13	1.3574 (14)
O1—C1	1.4570 (11)	C8—C9	1.4607 (14)
O2—C5	1.2148 (13)	C9—C10	1.5151 (13)
O3—C9	1.2345 (12)	C10—C11	1.5253 (15)
O4—C15	1.3718 (12)	C10—H10A	0.9700
O4—C20	1.4350 (12)	C10—H10B	0.9700
O5—C1	1.3962 (11)	C11—C12	1.5227 (14)
O5—H1O5	0.874 (18)	C11—H11A	0.9700
C1—C2	1.5203 (14)	C11—H11B	0.9700
C1—C6	1.5345 (14)	C12—C13	1.4982 (13)
C2—C3	1.5255 (14)	C12—H12A	0.9700
C2—H2A	0.9700	C12—H12B	0.9700
C2—H2B	0.9700	C14—C19	1.3931 (13)
C3—C4	1.5378 (16)	C14—C15	1.4091 (13)
C3—H3A	0.9700	C15—C16	1.3964 (13)
C3—H3B	0.9700	C16—C17	1.3961 (14)
C4—C5	1.5098 (15)	C16—H16A	0.9300
C4—H4A	0.9700	C17—C18	1.3884 (14)
C4—H4B	0.9700	C17—H17A	0.9300
C5—C6	1.5344 (13)	C18—C19	1.3976 (14)
C6—C7	1.5412 (14)	C18—H18A	0.9300
C6—H6A	0.9800	C19—H19A	0.9300
C7—C8	1.5133 (13)	C20—H20A	0.9600
C7—C14	1.5276 (13)	C20—H20B	0.9600
C7—H7A	0.9800	C20—H20C	0.9600
			
C13—O1—C1	117.22 (7)	C8—C9—C10	118.74 (9)
C15—O4—C20	116.87 (8)	C9—C10—C11	112.70 (8)
C1—O5—H1O5	106.7 (11)	C9—C10—H10A	109.1
O5—C1—O1	107.99 (7)	C11—C10—H10A	109.1
O5—C1—C2	112.32 (8)	C9—C10—H10B	109.1
O1—C1—C2	104.81 (7)	C11—C10—H10B	109.1
O5—C1—C6	108.38 (8)	H10A—C10—H10B	107.8
O1—C1—C6	109.47 (8)	C12—C11—C10	110.02 (8)
C2—C1—C6	113.68 (8)	C12—C11—H11A	109.7
C1—C2—C3	113.12 (9)	C10—C11—H11A	109.7
C1—C2—H2A	109.0	C12—C11—H11B	109.7
C3—C2—H2A	109.0	C10—C11—H11B	109.7
C1—C2—H2B	109.0	H11A—C11—H11B	108.2
C3—C2—H2B	109.0	C13—C12—C11	110.92 (8)
H2A—C2—H2B	107.8	C13—C12—H12A	109.5
C2—C3—C4	111.06 (9)	C11—C12—H12A	109.5
C2—C3—H3A	109.4	C13—C12—H12B	109.5
C4—C3—H3A	109.4	C11—C12—H12B	109.5
C2—C3—H3B	109.4	H12A—C12—H12B	108.0
C4—C3—H3B	109.4	O1—C13—C8	123.96 (9)
H3A—C3—H3B	108.0	O1—C13—C12	111.00 (8)
C5—C4—C3	109.20 (8)	C8—C13—C12	125.03 (9)
C5—C4—H4A	109.8	C19—C14—C15	117.89 (9)
C3—C4—H4A	109.8	C19—C14—C7	122.94 (8)
C5—C4—H4B	109.8	C15—C14—C7	119.10 (8)
C3—C4—H4B	109.8	O4—C15—C16	123.04 (9)
H4A—C4—H4B	108.3	O4—C15—C14	115.90 (8)
O2—C5—C4	122.38 (9)	C16—C15—C14	121.05 (9)
O2—C5—C6	122.26 (9)	C17—C16—C15	119.48 (9)
C4—C5—C6	115.35 (9)	C17—C16—H16A	120.3
C5—C6—C1	109.28 (8)	C15—C16—H16A	120.3
C5—C6—C7	111.86 (8)	C18—C17—C16	120.50 (9)
C1—C6—C7	112.48 (8)	C18—C17—H17A	119.8
C5—C6—H6A	107.7	C16—C17—H17A	119.8
C1—C6—H6A	107.7	C17—C18—C19	119.31 (9)
C7—C6—H6A	107.7	C17—C18—H18A	120.3
C8—C7—C14	113.17 (8)	C19—C18—H18A	120.3
C8—C7—C6	109.55 (8)	C14—C19—C18	121.74 (9)
C14—C7—C6	114.28 (8)	C14—C19—H19A	119.1
C8—C7—H7A	106.4	C18—C19—H19A	119.1
C14—C7—H7A	106.4	O4—C20—H20A	109.5
C6—C7—H7A	106.4	O4—C20—H20B	109.5
C13—C8—C9	118.53 (9)	H20A—C20—H20B	109.5
C13—C8—C7	122.45 (9)	O4—C20—H20C	109.5
C9—C8—C7	118.73 (8)	H20A—C20—H20C	109.5
O3—C9—C8	121.80 (9)	H20B—C20—H20C	109.5
O3—C9—C10	119.41 (9)		
			
C13—O1—C1—O5	71.99 (10)	C7—C8—C9—C10	−179.45 (8)
C13—O1—C1—C2	−168.09 (8)	O3—C9—C10—C11	−156.92 (9)
C13—O1—C1—C6	−45.81 (10)	C8—C9—C10—C11	25.45 (12)
O5—C1—C2—C3	−175.06 (8)	C9—C10—C11—C12	−53.49 (11)
O1—C1—C2—C3	67.95 (10)	C10—C11—C12—C13	50.03 (11)
C6—C1—C2—C3	−51.54 (11)	C1—O1—C13—C8	20.22 (13)
C1—C2—C3—C4	54.04 (11)	C1—O1—C13—C12	−160.09 (8)
C2—C3—C4—C5	−55.21 (12)	C9—C8—C13—O1	169.98 (8)
C3—C4—C5—O2	−122.06 (11)	C7—C8—C13—O1	−3.74 (15)
C3—C4—C5—C6	56.91 (11)	C9—C8—C13—C12	−9.66 (14)
O2—C5—C6—C1	125.76 (10)	C7—C8—C13—C12	176.62 (9)
C4—C5—C6—C1	−53.20 (11)	C11—C12—C13—O1	160.59 (8)
O2—C5—C6—C7	0.54 (13)	C11—C12—C13—C8	−19.73 (13)
C4—C5—C6—C7	−178.43 (8)	C8—C7—C14—C19	4.92 (14)
O5—C1—C6—C5	174.47 (8)	C6—C7—C14—C19	−121.40 (10)
O1—C1—C6—C5	−67.98 (10)	C8—C7—C14—C15	−171.98 (9)
C2—C1—C6—C5	48.83 (11)	C6—C7—C14—C15	61.70 (12)
O5—C1—C6—C7	−60.66 (10)	C20—O4—C15—C16	12.66 (14)
O1—C1—C6—C7	56.89 (10)	C20—O4—C15—C14	−168.10 (9)
C2—C1—C6—C7	173.70 (8)	C19—C14—C15—O4	−178.20 (9)
C5—C6—C7—C8	82.61 (9)	C7—C14—C15—O4	−1.14 (14)
C1—C6—C7—C8	−40.82 (10)	C19—C14—C15—C16	1.06 (15)
C5—C6—C7—C14	−149.21 (8)	C7—C14—C15—C16	178.12 (9)
C1—C6—C7—C14	87.36 (10)	O4—C15—C16—C17	177.27 (10)
C14—C7—C8—C13	−114.03 (10)	C14—C15—C16—C17	−1.94 (16)
C6—C7—C8—C13	14.77 (12)	C15—C16—C17—C18	1.30 (16)
C14—C7—C8—C9	72.27 (11)	C16—C17—C18—C19	0.17 (16)
C6—C7—C8—C9	−158.94 (8)	C15—C14—C19—C18	0.46 (15)
C13—C8—C9—O3	−170.97 (9)	C7—C14—C19—C18	−176.48 (9)
C7—C8—C9—O3	2.98 (14)	C17—C18—C19—C14	−1.07 (16)
C13—C8—C9—C10	6.60 (13)		

### Hydrogen-bond geometry (Å, °)

**Table:**

Cg1 is the centroid of the C14–C19 ring.

**Table:**

*D*—H···*A*	*D*—H	H···*A*	*D*···*A*	*D*—H···*A*
O5—H1O5···O3^i^	0.87 (2)	1.93 (2)	2.7877 (11)	166.3 (18)
C6—H6A···O4	0.98	2.32	2.9266 (12)	120
C16—H16A···O5^ii^	0.93	2.53	3.4172 (13)	160
C20—H20B···Cg1^ii^	0.96	2.67	3.5206 (13)	147

Symmetry codes: (i) *x*+1, *y*, *z*; (ii) −*x*+2, −*y*, −*z*+1.
